# Frequent Home Monitoring of ICD Is Effective to Prevent Inappropriate Defibrillator Shock Delivery

**DOI:** 10.1155/2014/579526

**Published:** 2014-01-29

**Authors:** Paolo Bifulco, Luigi Argenziano, Maria Romano, Mario Cesarelli, Mario Sansone, Stefano Casella, Stefano Nardi

**Affiliations:** ^1^DIETI, University of Naples “Federico II”, 80125 Naples, Italy; ^2^Presidio Ospedaliero Pineta Grande, Castel Volturno, 81030 Caserta, Italy

## Abstract

Recently, in the context of telemedicine, telemonitoring services are gaining attention. They are offered, for example, to patients with implantable cardioverter defibrillators (ICDs). A major problem associated with ICD therapy is the occurrence of inappropriate shocks which impair patients' quality of life and may also be arrhythmogenic. The telemonitoring can provide a valid support to intensify followup visits, in order to improve the prevention of inappropriate defibrillator shock, thus enhancing patient safety. Inappropriate shock generally depends on atrial fibrillation, supraventricular tachycardia, and abnormal sensing (such as those caused by electromagnetic interferences). As a practical example, an unusual case of an ICD patient who risked an inappropriate shock while taking a shower is reported. Continuous remote telemonitoring was able to timely warn cardiologist via GSM-SMS, who were able to detect improper sensing examining the intracardiac electrogram via Web. Patient was promptly contacted and warned to not further come in contact with the hydraulic system and any electrical appliance to prevent an inappropriate defibrillator shock. This demonstrates the effectiveness and usefulness of continuous remote telemonitoring in supporting ICD patients.

## 1. Introduction

In the last decades, the advances in information and communication technology have permitted that telehealth supports are diffusing rapidly into all aspects of healthcare. Integration of traditional medical practices with computerized supports and Internet functionalities may improve quality of life and solve problems of access health disparities for some patient categories [1–3].

Home-care is a term often used to describe telemedicine applications in which medical services are delivered to patients at their homes. Home-care is especially important for a specific group of patients with long-term chronic conditions, such as chronic cardiac diseases. Recently, there is a growing interest also in the remote monitoring and followup of implantable cardioverter defibrillators (ICDs). The number of patients with implantable devices has been growing steadily, mainly because indications for ICD shifted from secondary to primary prevention of sudden death [4] (for more details refer to Multicenter Automatic Defibrillator Implantation Trial II (MADIT II) [5]).

According to current guidelines (issued by AHA, ACC, etc.), patients with an ICD should be followed up every 3–6 months (increasing frequency as the battery approaches elective replacement) to ensure proper device function. However, a more frequent control could avoid bad workings of the device, so to guarantee a longer time-life of batteries. In particular, continuous telemonitoring of ICD can provide a valid support for followup and can potentially enhance also patient safety.

A major problem associated with ICD therapy is the occurrence of inappropriate shocks which impair patients' quality of life and may also be arrhythmogenic. In particular, arrhythmia episodes or oversensing that have led to delivered or aborted ICD therapies can be analyzed using telemonitoring and the opportune counter-measures considered. Indeed, inappropriate shocks for atrial arrhythmias with rapid ventricular conduction or for abnormal sensing result in multiple adverse effects including impaired quality of life, psychiatric disturbances, and even provocation of fatal ventricular arrhythmia. A recent study [6] reports that inappropriate shock occurred in 11.5% of the patients population considered; the inappropriate shocks resulted in 31.2% of the total shock episodes. Atrial fibrillation was the most common trigger for inappropriate shock (44%), followed by supraventricular tachycardia (36%), and then by abnormal sensing (20%), which can be due mainly to electromagnetic interferences.

Electromagnetic interference may cause erroneous recognition of ventricular fibrillation (VF) and be responsible of inappropriate shocks for implanted defibrillators [7–13]. A contact with an electrical device (e.g., an electrical appliance not correctly connected to ground, such as a washing machine or a refrigerator) can cause a serious passage of current inside the human body at power line frequency (50–60 Hz) that can interfere with the intracardiac electrograms (EGM) and simulate an episode of VF. In general, patients wearing an implantable stimulator are warned about this possible occurrence (direct and indirect contact with electrical equipment) whereas are not advised about contact with the hydraulic systems, which is not perceived by the patient as explicitly linked to the electrical risk, and so may hide a more insidious danger.

Major ICD factories supply their telemonitoring systems such as Home Monitoring (Biotronik); CareLink Network (Medtronic); Latitude Patient Management system (Boston Scientific); Merlin.net (St. Jude Medical). Remote pacemaker and ICD followup and monitoring system by Biotronik received the first FDA approval in 2001. The transmitter provided to each patient is a little bigger than a cell phone and communicates wirelessly with the ICD within a distance of 2 m. Once internal ICD data are received from the personal transmitter, they are automatically sent to a gathering centre using the GSM network. The choice of the GSM network does not force the patient to have a fixed phone or a DSL connection at home; this permits a great freedom of movement during daily life to the patient, ICD monitoring can take place continuously and does not require patient involvement. EGM of 30 s duration are sent periodically to the reference centre that may assist with data interpretation and are available to medical doctors via secured webpage on the Internet. Whenever critical data are available for consultation, the doctor is informed by one of the following modalities previously established: e-mail, SMS, fax, or phone messages. The types of events which trigger an alert can be customized for each patient.

## 2. Case Report: A Case of Power Line Interference with ICD during Showering

A 54-year-old man, suffering from hypertrophic obstructive cardiomyopathy, survived an intrahospital cardiac arrest. After this episode, a transvenous ICD system (Biotronik Lumos DRT) equipped with atrial and ventricular electro catheter system (Biotronik) was implanted. Subsequent followup visits proved normal functioning of the ICD device, no arrhythmias episodes were detected, and no defibrillator discharges were reported.

The patient was continuously monitored via the Biotronik Home-Monitoring system described above.

**Figure 1:**
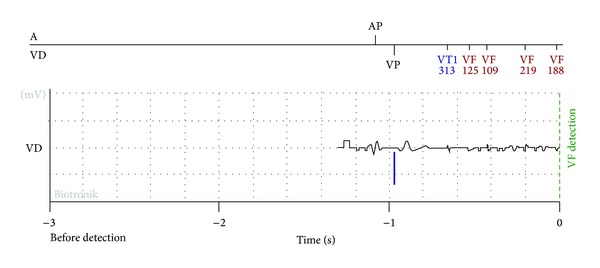
Intracardiac EGM as available from the website of the home-care ICD telemonitoring system.

About one year after ICD implantation the cardiologist of the centre of electrophysiology, who is in charge of the patient, received an alert of VF detection from that patient via GSM. Cardiologist promptly consulted the data concerning the arrhythmias episode and the relative intracardiac EGM via the website (see Figure 1).

**Figure 2:**
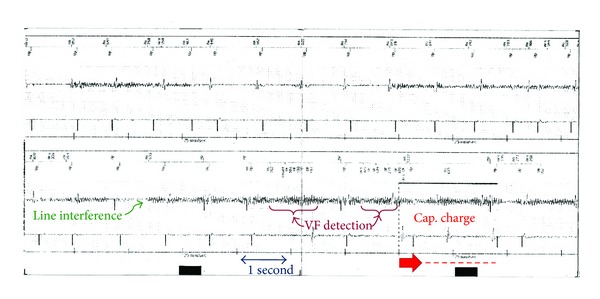
Detail of the intracardiac EGM atrium and ventriculum. Power line interferences clearly visible on EGM (thickening of signal); it followed VF detection (purple arrows) and the start of the capacitor charge (red arrow).

An episode of ventricular tachycardia (VT) was documented followed by VF detection, a ventricular oversensing, likely due to power line interference, which appeared from the EGM. Power line artifacts were present of both atrial and ventricular sensing channels resulting in inhibition of the antibradycardia pacing functions and in some cases in ventricular pacing due to atrial oversensing (see Figure 2).

However, after charging of the capacitor, no shock was actually delivered because the interference paused just before reconfirmation of VF arrhythmia.

As soon as possible, the patient was contacted and was interviewed about the episode. Specific radiographic examinations have excluded fracture (even incomplete) of the ICD leads. Accurate impedance tests of the ICD leads have also excluded isolation defects. The patient reported no preceding or accompanying symptoms of palpitations, light-headedness, or syncope. During patient consultation, thanks to the log time of the episode, it was assessed that the patient was likely taking a shower during the false arrhythmia detection. Therefore, the patient was warned to not be in contact with the shower neither with the hydraulic system, waiting for a proper maintenance of the electrical and hydraulic plants.

Afterwards, a technical inspection took place at the patient's site. Once the electrocution via the showering apparatus probably due to improper grounding of electrical appliances was confirmed, a technical maintenance company was contacted to verify and mend the plants.

## 3. Discussion

Telemedicine is gradually becoming a technological and clinical reality and is covering different medicine branches. The growing number of patients with ICD indication, involving not only cardiac patients [14], and the high complexity of modern devices thrust for efficient therapy surveillance. The capability of telemedicine personal patient device to automatically and rapidly transfer information with the implanted device opens new scenarios in ICD patient management.

The employment of mobile phone network does not restrict patient monitoring at home but can be effectively used ubiquitously. No-involvement of patient in emergency cases can be also a favorable feature.

Real-time telemedicine has greater overheads than store-and-forward approaches but permits immediate interaction. However for ICD routine followup the store-and-forward approach can be effective and potentially decrease costs while enhancing patient safety.

Home monitoring bears the potential to reduce the need for routine in clinic ICD followup, in which the majority of cases reveal no need for interference [15] but cumulatively require significant personal and financial resources. The current practice of regular ICD controls has to be reconsidered in view of the growing ICD population [16–18].

This report described a case in which, thanks to the frequent ICD telemonitoring, the patient was promptly advised about a danger due to unexpected current leaks through the hydraulic plant, which caused an inappropriate VF detection when taking a shower. Inappropriate defibrillator shock was avoided through telemedicine, whereas classical health care delivery probably would never have been able to detect this trouble.

Future development of dedicated guidelines could constitute a key step to improve the safety, consistency, and efficiency of this ICD telemonitoring activity. Also ethical and legal aspects still remain largely unaddressed.
